# Possibly Abortive Cerebro-Spinal Fever

**Published:** 1915-09-18

**Authors:** 


### IN A GENERAL HOSPITAL.

### POSSIBLY ABORTIVE CEREBRO-SPINAL FEVER.

Since the mobilisation of August 1914 there have been, as we know, outbreaks in England of cerebro-spinal fever, one of those diseases which are familiar in text-books of medicine but are rarely seen in ordinary practice. Cerebro-spinal fever is *par excellence* a disease of armies and camps: although spasmodic outbreaks of it do now and then occur in peace-time, it is seldom that an epidemic of any considerable size is seen. From the accounts published in the medical Press during the last few months, it is evident that the classical picture of the disease as drawn in text-books is often departed from, and that the diagnosis instead of being simple may be most difficult: many of the typical symptoms—petechial rash, for instance, and head retraction—may be absent throughout the course of the illness. In suggesting the possibility that the cases I am about to relate may have been abortive and atypical cerebro-spinal fever, I am well aware that no strict proof of the hypothesis can be offered: I merely present it for what it is worth, without dogmatising one way or the other.

### Summary of the Cases.

It will, perhaps, be best if I detail in chronological order the four cases as they were seen. The salient features of the cases can be briefly summarised before I enter into the rather extensive history and multitude of details of each individual case. The first case was one of severe pyrexia of intermittent type, lasting six or seven weeks, associated with recurring erythematous rashes, which came out every three or four days. This rash was very profuse, and affected the extensor more than the flexor surfaces of the limbs; it was also present, less profusely, on the face, neck, and back, though but little of it appeared on the front of the trunk. Malaria, enteric fever, and rheumatism seemed to be satisfactorily put out of court. A modified Kernig's sign, exaggerated knee jerks, with ankle clonus, and on one occasion unequal pupils, were the only signs of disturbance in the nervous system. Profuse daily sweats were a prominent feature.

The second case was that of a French soldier, whose symptoms and signs for the first three or four weeks were an exact counterpart of those of the first case. In this case, however, there eventually developed stiff neck, purpura, ptosis, vomiting, and intense headache, followed by complete recovery. This case gave me the clue to the first one, and led me into a suspicious mood, which resulted in the early detection and isolation of cases three and four. In these two cases a very similar initial history and list of physical signs were present; they were both promptly submitted to lumbar puncture, and although no specific organism was obtained from the cerebro-spinal fluid, the symptoms of illness almost immediately vanished. In other words, lumbar puncture was a dead failure as a diagnostic measure, but appears to have been a great success as a therapeutic one.

